# Formation of gold nanoparticles by glycolipids of *Lactobacillus casei*

**DOI:** 10.1038/srep34626

**Published:** 2016-10-11

**Authors:** Fumiya Kikuchi, Yugo Kato, Kazuo Furihata, Toshihiro Kogure, Yuki Imura, Etsuro Yoshimura, Michio Suzuki

**Affiliations:** 1 Department of Applied Biological Chemistry, Graduate School of Agricultural and Life Sciences, The University of Tokyo, 1-1-1 Yayoi, Bunkyo-ku, Tokyo 113-8657, Japan; 2 Department of Earth and Planetary Science, Graduate School of Science, The University of Tokyo, 7-3-1 Hongo, Bunkyo-ku, Tokyo 113-0033, Japan; 3 The Open University of Japan, 2-11 Wakaba, Mishima-ku, Chiba-city, Chiba, 261–8586, Japan

## Abstract

Gold nanoparticles have particular properties distinct from those of bulk gold crystals, and such nanoparticles are used in various applications in optics, catalysis, and drug delivery. Many reports on microbial synthesis of gold nanoparticles have appeared. However, the molecular details (reduction and dispersion) of such synthesis remain unclear. In the present study, we studied gold nanoparticle synthesis by *Lactobacillus casei*. A comparison of *L. casei* components before and after addition of an auric acid solution showed that the level of unsaturated lipids decreased significantly after addition. NMR and mass spectrum analysis showed that the levels of diglycosyldiacylglycerol (DGDG) and triglycosyldiacylglycerol (TGDG) bearing unsaturated fatty acids were much reduced after formation of gold nanoparticles. DGDG purified from *L. casei* induced the synthesis of gold nanoparticles *in vitro*. These results suggested that glycolipids, such as DGDG, play important roles in reducing Au(III) to Au(0) and in ensuring that the nanoparticles synthesized remain small in size. Our work will lead to the development of novel, efficient methods by which gold nanoparticles may be produced by, and accumulated within, microorganisms.

Gold nanoparticles (containing a few tens of gold atoms) have various unique properties^1^,^2^. A gold nanoparticle solution is wine-red in color, because of surface plasmon resonance (SPR). Gold nanoparticles of various colors have been used by artists for several centuries. Over the past two decades, such nanoparticles have found novel applications in general industry, chemistry, biology, and medicine. Antibody-bearing nanoparticles are useful labeling agents in electron microscopy, and can reveal the detailed locations of organic molecules within cell organelles^3^. Gold nanoparticles attached to DNA fragments can detect DNA-DNA interactions (which trigger color changes)^4^. This technology is used to diagnose viral infections^5^,^6^. When synthesizing gold nanoparticles, the appropriate selections of reducing agents active on gold ions, and dispersing agents that hold the particle size in the nanometer range, are important. Various reducing and dispersing agents have been developed^7^,^8^,^9^,^10^. Industrially, large amounts of gold nanoparticles are synthesized in the reaction of gold with citric acid under conditions of high temperature and pressure^11^. Novel methods using macromolecular polymers or biomacromolecules, such as proteins and DNA, have been used to develop more functional gold nanoparticles (controlled in terms of shape) at low financial and energy costs, in the absence of unwanted byproducts^12^,^13^,^14^,^15^. Recently, many methods using microorganisms to synthesize metallic nanoparticles have been reported. One previous study showed that bacteria could induce the formation of magnetite crystals, reduce Ag^+^ and Au^3+^ ions to metal nanoparticles, and form ceramic/metal composites^16^,^17^,^18^,^19^,^20^. Algae have been used to synthesize the semiconducting particles (such as CdS) called quantum dots^21^. Fungi can also produce nanoparticles of Ag, Au, and CdS^22^,^23^. In such processes, the microorganisms are cultured at normal temperature under normal pressure and do not produce any toxic byproduct. Such benefits suggest that the use of microorganisms to synthesize gold nanoparticles will be important in the future. To improve the efficiency of such synthesis, it is essential to clarify the molecular mechanisms involved. An earlier report showed that *Lactobacillus* strains could produce gold nanoparticles^24^. However, no organic molecule relevant to such synthesis has yet been identified. In the present study, we extracted and identified organic molecules important in terms of the synthesis of gold nanoparticles by *L. casei*.

## Results

### Reaction of auric acid solution with *L. casei* cells

**Figure 1:**
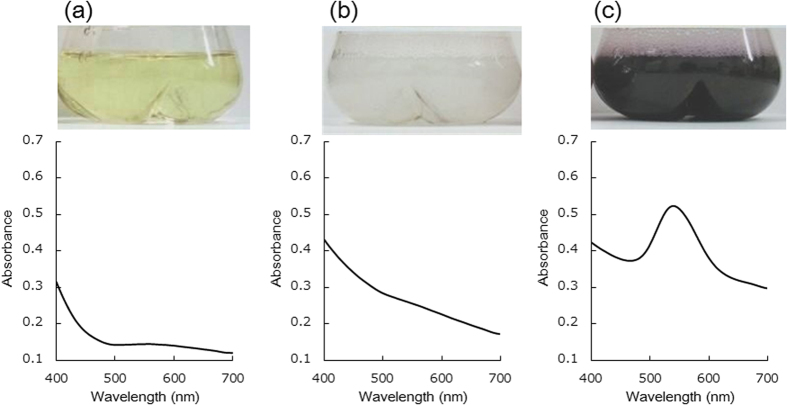
(**a**) Auric acid solution (0.5 mM K[AuCl_4_]) and its UV/VIS spectrum after 24 h. (**b**) The solution suspended by *L. casei* cells (2.0 g/L) and its UV/VIS spectrum after 24 h. (**c**) The solution containing both *L. casei* cells (2.0 g/L) and auric acid (0.5 mM K[AuCl_4_]) and its UV/VIS spectrum after 24 h.

Concentrated *L. casei* cells (2.0 g/L) were washed with distilled water to remove the culture medium and incubated in an auric acid solution (0.5 mM K[AuCl_4_]) for 24 h. The colors of the control auric acid solution and *L. casei* suspension were transparently yellow and turbidly white, respectively, at this time (Fig. 1a,b). The suspension containing both *L. casei* cells and auric acid was violet in color after 24 h (Fig. 1c). The suspensions were next subjected to ultrasonication and a UV/VIS spectroscope was used to measure absorbance at wavelengths from 400 to 700 nm. The violet suspension had an absorption maximum at 534 nm (Fig. 1c). However, not all suspensions of *L. casei* cells mixed with auric acid were violet in color (Fig. S1). A combination of *L. casei* cells (1.0 g/L) and auric acid (0.25 mM K[AuCl_4_]) was indeed violet; but a combination of *L. casei* cells (2.0 g/L) and auric acid (0.25 mM K[AuCl_4_]) remained white, indicating that an excess of *L. casei* cells inhibited development of the violet color. A combination of *L. casei* cells (0.25 g/L) and auric acid (0.25 mM K[AuCl_4_]) was violet, but a combination of *L. casei* cells (0.25 g/L) and auric acid (0.5 mM K[AuCl_4_]) yielded a dark precipitate, indicating that excess auric acid also inhibited development of the violet color. These results suggested that the violet color developed only when the ratio of *L. casei* cells to auric acid was appropriate. Therefore, we mixed auric acid (0.5 mM K[AuCl_4_]) and *L. casei* cells (2.0 g/L) to prepare a violet suspension [the Au(+) condition]; a suspension of *L. casei* cells only (2.0 g/L) served as the negative control [the Au(−) condition].

### Identification of gold nanoparticles

**Figure 2:**
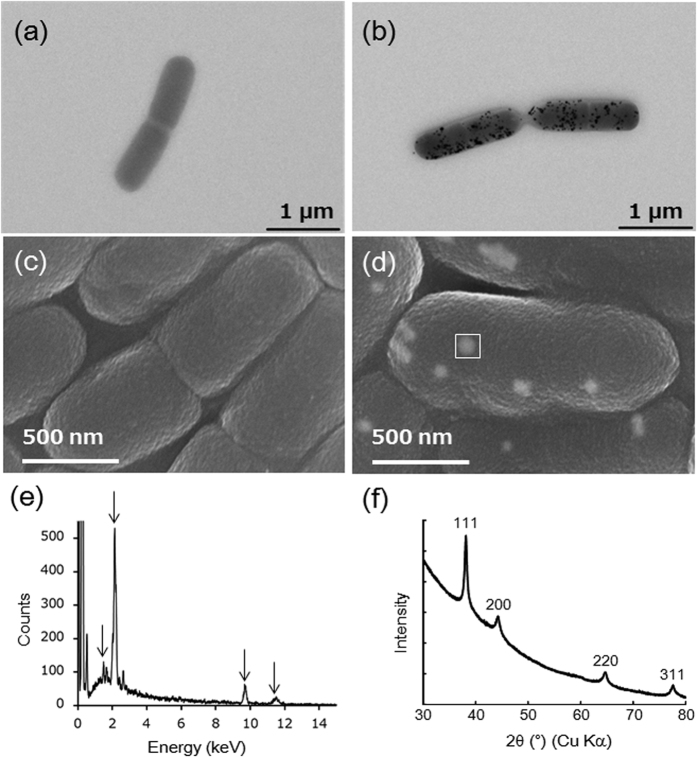
(**a**,**b**) TEM images of *L. casei* cells in (**a**) Au(−) and (**b**) Au(+). (**c**,**d**) SEM micrographs of *L. casei* cells in (**c**) Au(−) and (**d**) Au(+). The white square in (**d**) indicates the area for EDS analysis in (**e**). (**e**) EDS from the squared area in (**d**). The arrows showed the characteristic X-ray spectra of Au. (**f**) XRD pattern from the dried *L. casei* cells in Au(+).

To explore why the violet color developed, we subjected both Au(−) and Au(+) *L. casei* samples to both transmission and scanning electron microscopy (TEM and SEM, respectively). The Au(−) *L. casei* cells were baculiform in shape (1.9 μm in length, 0.4 μm in width) (Fig. 2a). However, many nanoparticles (7–56 nm in diameter) were evident in Au(+) cells (Fig. 2b). The sizes of synthesized particles were measured by ImageJ. The size value of the highest frequency was about 30 nm. The size distribution graph was described in Fig. S2. Similarly, SEM of Au(−) *L. casei* showed only cells that were baculiform in shape (Fig. 2c), whereas the *L. casei* Au(+) cells had many bright surface-attached nanoparticles (Fig. 2d). Energy dispersion spectroscopy (EDS) showed that the nanoparticles were composed of Au (Fig. 2e). X-ray diffraction (XRD) measurements on dried *L. casei* Au(+) cells revealed several peaks corresponding to gold crystals with a face-centered cubic structure (Fig. 2f). These results indicated that *L. casei* cells mixed with an auric acid solution produced gold nanoparticles. As in liquid suspension, the gold nanoparticles absorbed light of wavelengths between 500–600 nm via surface plasmon resonance (SPR), the violet color of the mixture of *L*. *casei* with auric acid was attributable to formation of such nanoparticles.

### Identification of key organic molecules in *L. casei* for the synthesis of gold nanoparticles

**Figure 3:**
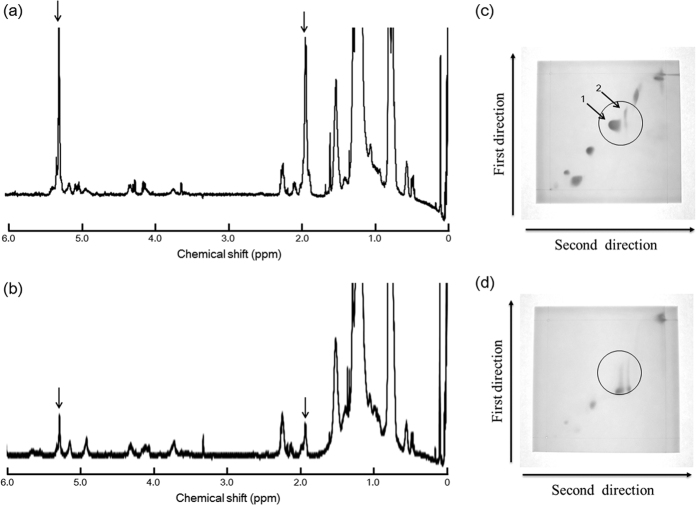
(**a**) NMR spectrum of Au(−) extract. (**b**) NMR spectrum of Au(+) extract. (**c**) TLC analysis of Au(−) extract. (**d**) TLC analysis of Au(+) extract.

After ultrasonication, the supernatants of *L. casei* Au(−) and Au(+) cells were turbidly white with an extreme meniscus and transparently violet without an extreme meniscus, respectively (Fig. S3). We assumed that these differences in physical properties were probably attributable to differences in lipid composition. *L. casei* Au(−) and Au(+) cells were suspended in chloroform/methanol solution (2:1, v/v) to extract lipids, and these extracts were subjected to [^1^H]-NMR analysis. Comparison of the [^1^H]-NMR spectra showed that the heights of the 1.94- and 5.35-ppm peaks were significantly lower in the Au(+) than the Au(−) extract (Fig. 3a,b), suggesting that particular lipids may have been associated with gold nanoparticle synthesis. We next subjected both lipid extracts to two-dimensional thin-layer chromatography (TLC). A comparison of the spot patterns showed that two distinct spots (1 and 2, circled in Fig. 3c,d) in the Au(−) extract were fuzzy, smeared, and tailed in the Au(+) extract.

**Figure 4:**
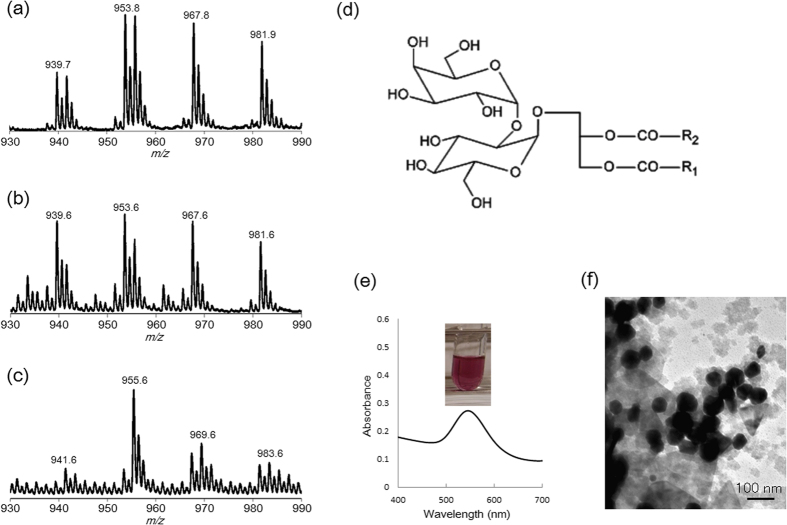
(**a**) MALDI-TOF-MS spectrum of the extract from spot 1 in Fig. 3(c). (**b**) MALDI-TOF-MS analysis of Au(−) extract. (**c**) MALDI-TOF-MS analysis of Au(+) extract. (**d**) The schematic structure of DGDG. (**e**) The solution containing both DGDG (5 μg/L) and auric acid (0.25 mM K[AuCl_4_]) and its UV/VIS spectrum after 24 h. (**f**) TEM image of gold nanoparticles synthesized by DGDG.

To identify the lipids of spots 1 and 2, the silica gel regions bearing these spots were scraped off the TLC plate, the lipids extracted was dissolved into a chloroform/methanol solution (2:1, v/v), and subjected to MS analyses. MALDI-TOF MS showed that spot 1 contained four major peaks at 939.6, 953.6, 967.6, and 981.6 (*m*/*z*), and certain isotope peaks (Fig. 4a). These peaks were also evident in the Au(−) *L. casei* extract (Fig. 4b). However, especially in the region around 930–990 (*m*/*z*), the peak profiles differed dramatically (Fig. S4). The intensities of all such peaks (*m*/*z*) were reduced in the Au(+) extract. Four major peaks [at 941.6, 955.6, 969.3, and 983.6 (*m*/*z*)] were detected (Fig. 4c). In the Au(+) extract, the heights of these four peaks were twice those of the Au(−) extract. MS analyses (ESI-TOF-MS spectroscopy and MALDI-TOF/TOF MS spectroscopy) revealed that the four major peaks at 939.6, 953.6, 967.6, and 981.6 (*m*/*z*) corresponded to diglycosyldiacylglycerol (DGDG) with two unsaturated fatty acids, the carbon chains of which differed in length (Fig. S5, Fig. 4d, and Table 1). Two larger masses (around *m*/*z* 969.3) detected in Au(+) *L. casei* also corresponded to DGDG, but with one saturated and one unsaturated fatty acid (Fig. S5 and Table 1).

NMR spectrum analyses revealed the structures of the DGDG saccharides. H1-6 and H1′-6′ indicate the protons of galactose and glucose, respectively. C1-6 and C1′-6′ indicate the carbons of galactose and glucose, respectively. The DQF-COSY spectrum revealed anomeric protons at 4.96 ppm (Fig. S6). The 4.96 ppm signal had two correlations, suggesting that two anomeric protons from two monosaccharides overlapped. The signals at 5.2 ppm are from two protons of carbons bound to the glycerol ester. The signal at 5.3 ppm is from a proton of a carbon bound to the glycerol ether. DQF-COSY signal assignment also revealed the signals from H1-5 of galactose and H1′-H4′ of glucose. The HSQC spectrum revealed all carbons of galactose and glucose (Fig. S7). The HMBC spectrum revealed a correlation between C1 and H2′, suggesting that carbon 1 of galactose was bound to carbon 2′ of glucose (Fig. S8). We also noted a correlation between C1′ and the glycerol protons, suggesting that carbon 1′ of glucose was bound to glycerol (Fig. S9). These results suggested that DGDG was 1,2-di-*O*-acyl-3-*O*-[*O*-α-D-galactopyranosyl-(1 → 2)-α-D-glucopyranosyl]glycerol (Fig. S9). On the other hand, MALDI-TOF MS analysis showed that the extract of spot 2 contained four major peaks at 1,101.2, 1,115.2, 1,129.2, and 1,143.2 (*m*/*z*), and certain isotope peaks (Fig. S10). The intensities of these four peaks were reduced in the chloroform/methanol extract of *L. casei* Au(+) cells. MS/MS and high-resolution MS analyses revealed that the four major peaks at 1,101.2, 1,115.2, 1,129.2, and 1,143.2 (*m*/*z*) corresponded to triglycosyl diacylglycerol (TGDG) bearing two unsaturated fatty acids, the carbon chains of which differed in length (Fig. S11 and Table 1).

### Synthesis of gold nanoparticles using purified DGDG

DGDG extracted from the silica gel around spot 1 was solubilized in ethanol, mixed with auric acid, and incubated at 37 °C for 24 h. At this time, the color of the solution (5 mg/L, 10 mg/L and 20 mg/L DGDG) became violet (Fig. 4e, Fig. S12). The absorption maximum varied by the DGDG concentration. TEM showed that gold nanoparticles were synthesized (Fig. 4f). The diameter of the gold nanoparticles formed was slightly greater than that of nanoparticles synthesized by *L. casei* cells. The size value of the highest frequency was about 60 nm (Fig. S12b). On the other hand, the commercially available purified DGDG (caDGDG: 1,2-diacyl-3-*O*-[(α-D-galactosyl(1-6)-β-D-galactosyl]glycerol with two chains of fatty acids (C16:1 and C20:5))) was also used for the formation of gold nanoparticles. The color of the solution (5 mg/L, 10 mg/L and 100 mg/L caDGDG) also became violet. The absorption spectra of caDGDG were very similar to those of purified DGDG from TLC spot. TEM observation showed that gold nanoparticles were synthesized (Fig. S13). The size value of the highest frequency was about 60 nm (Fig. S13c). These results suggested that the glycolipid such as DGDG is one of the key factors for the formation of gold nanoparticles in *L. casei*.

## Discussion

Although many previous studies have shown that *L. casei* cells produce gold nanoparticles, the molecular mechanism remains unknown. In the present study, we sought to identify organic molecules involved in gold nanoparticle synthesis.

In the previous report, gold nanoparticles formed in the culture medium^24^. However, we found that such synthesis was unreliable, because the culture medium contains many components either inhibiting or promoting nanoparticle synthesis, and such synthesis varied by the growth stage of *L. casei*. To identify the key organic molecules involved, we found it necessary to remove the culture medium. Therefore, we washed the cells with distilled water and then mixed the washed cells with auric acid. We found that the ratio of *L. casei* cell numbers to auric acid concentration was important in terms of nanoparticle synthesis. Excess auric acid inhibited *L. casei* metabolism, and an excessive number of *L. casei* cells resulted in the production of large amounts of reducing agents, which may induce polymerization of certain cellular components, in turn inhibiting gold nanoparticle synthesis.

DGDG and TGDG were involved in gold nanoparticle synthesis by *L. casei*. These glycolipids are probably components of the membrane lipid bilayer, replacing phospholipids; glycolipid synthesis requires less energy than phospholipid synthesis. Therefore, DGDG and TGDG of *L. casei* are probably present in the cell membrane^25^,^26^,^27^. TEM and SEM showed that gold nanoparticles formed on the cell surface, suggesting that the reduction reaction [Au(III) to Au(0)] occurred on the cell membrane containing DGDG and TGDG. After nanoparticle synthesis, most DGDG and TGDG with two unsaturated fatty acids disappeared; small amounts of DGDG and TGDG containing single unsaturated fatty acids remained. One previous report also found that unsaturated fatty acids present in vegetable oil reduced metal ions and bound to the surface of nanoparticles^28^. Therefore, the unsaturated bonds in the fatty acids of DGDG and TGDG may be associated with reduction of Au(III).

Kumar *et al*.^28^, suggested that the hydrogen in the allylic position of an unsaturated fatty acid can be desorbed by light or an oxidizing agent, creating a fatty acid radical^28^. The electron of the hydrogen radical may be transferred to Au(III) in a reducing reaction. Au(III) then becomes the unstable Au(II); two Au(II) ions become an Au(III) and an Au(I) ion via a disproportionation reaction. Au(I) then receives an electron from a hydrogen radical to become Au(0). On the other hand, autoxidation creates lipid peroxides in DGDG and TGDG; these are ultimately decomposed in a radical chain reaction. According to this mechanism, the speculative autoxidation process for the formation of gold nanoparticles was described in Fig. S14. The reaction between glycolipids containing unsaturated fatty acids (GLU) and Au(III) is shown below:


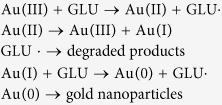


In the present study, incubation of DGDG with auric acid *in vitro* resulted in production of gold nanoparticles, suggesting that DGDG functioned not only as a reducing agent, but also as a dispersing agent. The carboxylic groups of citric acid bind to the surface of Au(0) to create nanoparticles^11^. The carboxylic groups of the DGDG fatty acids may interact with the Au(0) surface to stabilize nanoparticles. However, the gold nanoparticles synthesized from DGDG were slightly larger than those synthesized by *L. casei* cells, suggesting that any dispersing capacity of DGDG may be limited; other (as yet unknown) dispersing agents within *L. casei* cells may also play roles in inhibiting Au(0) aggregation and crystal growth. Further work is thus needed to reveal exactly how *L. casei* forms gold nanoparticles. Identification of the key organic molecules may allow modification of *L. casei* genes, permitting such recombinants to produce gold nanoparticles more effectively. In the future, *L. casei* strains modified along these lines may find applications in metal recycling and phytoremediation.

## Methods

### Synthesis of gold nanoparticles by *L. casei*

*L. casei* (strain JCM1134) was purchased from RIKEN Microbe Division. *L. casei* was cultured in the MRS liquid medium (1% casein peptone, tryptic digest/1% meat extract/0.5% yeast extract/2% glucose/0.1% tween80/0.2% K_2_HPO_4_/0.5% CH_3_COONa/0.2% diammonium citrate/0.02% MgSO_4_ · 7H_2_O/0.005% MnSO_4_ · 5H_2_O) at 37 °C for 8 h of preculture and for 14 h of main culture with shaking (300 rpm). The correlation between OD_600_ and dry bacterial cell weight was calculated to estimate the amounts of bacterial cells in the solution (Fig. S15). The bacterial cells were centrifuged and washed by distilled water to remove the liquid medium. The precipitation of bacterial cells was suspended in various concentrations of auric acid solution (K[AuCl_4_]). The mixture solution of bacterial cells and auric acid were incubated on the shaker (180 rpm) at 37 °C for 24 h.

### UV/VIS spectroscopy

The mixture solutions of bacterial cells and auric acid after 24 h were treated with ultrasonication (U200S control, IKA LABOTECH INK) to break the cell. After centrifugation to remove the precipitation, UV/VIS spectra of 10 diluted supernatant were measured at the range of wavelength from 400 to 700 nm using UV/VIS spectroscopy photometer (V-550 spectrophotometer, JASCO). The mixture solutions of purified DGDG (DGDG from TLC plate and caDGDG) and auric acid after 24 h were also collected. 10 diluted solutions were measured at the range of wavelength from 400 to 700 nm using UV/VIS spectroscopy photometer.

### Transmission and scanning electron microscopies (TEM and SEM)

To examine the origin of violet color, TEM observations of *L. casei* cells in Au(−) and Au(+) were performed for several representative specimens. TEM analyses were performed using a JEOL JEM-2000EX TEM operated at 200 kV. SEM observations were carried out using an S-4800 SEM (HITACHI) with a cold field-emission gun at 15 kV. Specimens for SEM were coated with carbon before observations. X-ray microanalysis was performed using energy-dispersive spectroscopy (EDS) with an ultra-thin window detector (HORIBA) equipped to the SEM. The particle sizes observed by TEM were measured by ImageJ. The bigger precipitates more than 100 nm were removed from the counts.

### X-ray diffraction (XRD) measurements

The dried powders of *L. casei* cells incubated in auric acid solution were filled up in a nonreflecting silicon sample holder for XRD. XRD patterns were collected using a RINT-Ultima^+^ diffractometer (RIGAKU) with CuKα radiation emitted at 40 kV and 30 mA.

### Extraction of lipid from *L. casei*

The extraction of lipid was performed according to the Bligh & Dyer method^29^. Chloroform/methanol (2:1) solution was added to the precipitation of bacterial cells with or without the treatments of auric acid solution. After suspension and centrifugation, chloroform layer was extracted to remove the water layer and precipitation using the 22G syringe (TERUMO). For the NMR analyses, the deuterated chloroform/methanol instead of normal chloroform/methanol was used to extract the lipid from *L. casei*.

### Nuclear magnetic resonance (NMR) spectroscopy

The samples were dissolved in deuterated chloroform/methanol (1:1). One- and two-dimensional NMR spectra were measured at 20 °C on a JMN-A500 (resonance frequency of ^1^H: 500 MHz) spectrometer (JEOL). The ^1^H, ^1^H-COSY spectra were measured with 512 points in f2, 2048 points in f1, a 45016.66 Hz spectral width in f2, a 5628.1 Hz spectral width in f1. The ^13^C, ^1^H-HSQC spectra were measured with 256 points in f2, 1024 points in f1, a 21385.8 Hz spectral width in f2, a 6256.26 Hz spectral width in f1. The ^13^C, ^1^H-HMBC spectra were measured with 512 points in f2, 2048 points in f1, a 25176.23 Hz spectral width in f2, a 6256.26 Hz spectral width in f1. As an internal standard compound, tetramethylsilane (0 ppm, Wako) was used for ^1^H-NMR and ^13^C-NMR.

### Two dimensional thin layer chromatography (TLC) analysis

In the first dimension, after spotting of samples in the origin point, plates were transferred to TLC chambers saturated with the chromatographic solvent (Chloroform/methanol/acetic acid, 65:25:10). In the second dimension, the other side was put in the bottom by tilting the plates with 90 degrees and transferred to another TLC chambers saturated with the chromatographic solvent (Chloroform/methanol/formic acid, 65:25:10). After drying, the plates were incubated in the bottle with iodine grains to detect the unsaturated lipids.

### MS analysis

Mass spectra were measured on a matrix-assisted laser-desorption ionization–time-of-flight mass spectrometer (ultraflex MALDI-TOF/TOF, Bruker). The sample solution was mixed with 500 mM 2,5-dihydroxybenzoic acid in chloroform/methanol (1:1) solution as the matrix and dried on the plate. High resolution mass spectra were measured on a time-of-flight mass spectrometer JMS-T100LC AccuTOF (JEOL) equipped with an electrospray ionization source in the positive ion mode. The extracted samples were dissolved in methanol and applied to the mass spectrometer. The settings of the instrument were calibrated using sodium trifluoroacetate. The ion-source temperature was 250 °C. The mass analyzer was scanned from m/z 300 to 1500 for the full scan analysis.

### Extraction of diglycosyldiacylglycerol (DGDG)

The spot of TLC analysis containing DGDG was scraped from the surface of thin chromatography layer. DGDG was extracted from the powder of silica gel using chloroform/methanol solution (2:1). The extract was evaporated to concentrate DGDG. The purified DGDG powder was dissolved in ethanol.

### Synthesis of gold nanoparticles using purified DGDG

DGDG extraction from the TLC plate dissolved in 40 μl ethanol was applied to 960 μl of auric acid solution (final concentration of K[AuCl_4_]: 250 μM). The mixture solution of DGDG and auric acid was incubated at 37 °C for 24 h. 40 μl of ethanol without DGDG was mixed with 960 μl of auric acid solution (final concentration of K[AuCl_4_]: 250 μM) as a negative control experiment. UV/VIS spectra of the supernatant were measured and the synthesized gold nanoparticles were observed by TEM. On the other hand, we used the commercially available purified DGDG (caDGDG) for the same experiment described above. caDGDG is 1,2-diacyl-3-*O*-[(α-D-galactosyl(1-6)-β-D-galactosyl]glycerol with two chains of fatty acids (C16:1 and C20:5) (Carbosynth).

## Additional Information

**How to cite this article**: Kikuchi, F. *et al*. Formation of gold nanoparticles by glycolipids of *Lactobacillus casei*. *Sci. Rep.* **6**, 34626; doi: 10.1038/srep34626 (2016).

## Supplementary Material

Supplementary Information

## Figures and Tables

**Table 1:** Structures of glycolipids.

*m*/*z*	Number of glycosyl structure	Fatty acids
939.6	2	C16:1, C18:1
953.6	2	C16:1, C19:1
967.6	2	C18:1, C18:1
981.6	2	C18:1, C19:1
969.3	2	C17:0, C19:1
1101.2	3	C16:1, C18:1
1115.2	3	C16:1, C19:1
1129.2	3	C18:1, C18:1
1143.2	3	C18:1, C19:1
