# Author Correction: Formation of gold nanoparticles by glycolipids of *Lactobacillus casei*

**DOI:** 10.1038/s41598-020-63177-3

**Published:** 2020-04-20

**Authors:** Fumiya Kikuchi, Yugo Kato, Kazuo Furihata, Toshihiro Kogure, Yuki Imura, Etsuro Yoshimura, Michio Suzuki

**Affiliations:** 1 Department of Applied Biological Chemistry, Graduate School of Agricultural and Life Sciences, The University of Tokyo, 1-1-1 Yayoi, Bunkyo-ku, Tokyo 113-8657 Japan; 2 Department of Earth and Planetary Science, Graduate School of Science, The University of Tokyo, 7-3-1 Hongo, Bunkyo-ku, Tokyo 113-0033 Japan; 3 The Open University of Japan, 2-11 Wakaba, Mishima-ku, Chiba-city, Chiba, 261–8586 Japan

Correction to: *Scientific Reports* 10.1038/srep34626, published online 11 October 2016

This Article contains errors in the Supplementary Information file, where the size distributions of the nanoparticles given in Figure S12b and Figure S13c are incorrect. The correct Figures S12 and S13 are given below as Figs. 1 and 2 respectively.

**Figure 1:**
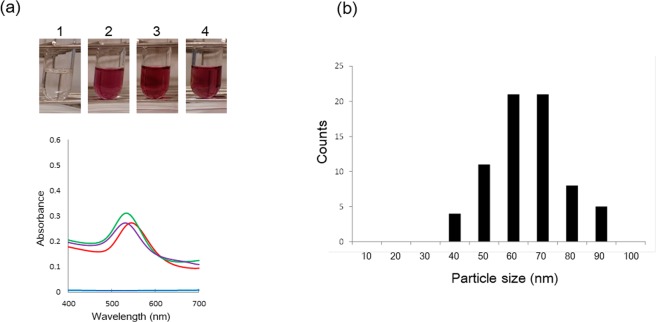
(**a**) Auric acid solution (0.25 mM K[AuCl4]) with DGDG purified from theTLC plate (1: 0 μg/mL, 2: 5.0 μg/mL, 3: 40 μg/mL, 4: 160 μg/mL) and their UV/VIS spectra after 24 h. The blue line: 0 mg/mL DGDG, the red line: 5.0 μg/mL DGDG, the green line: 40 μg/mL DGDG, the purple line: 160 μg/mL in UV/VIS spectra. (**b**) The size distribution of nanoparticles in the image of Fig. 4f.

**Figure 2:**
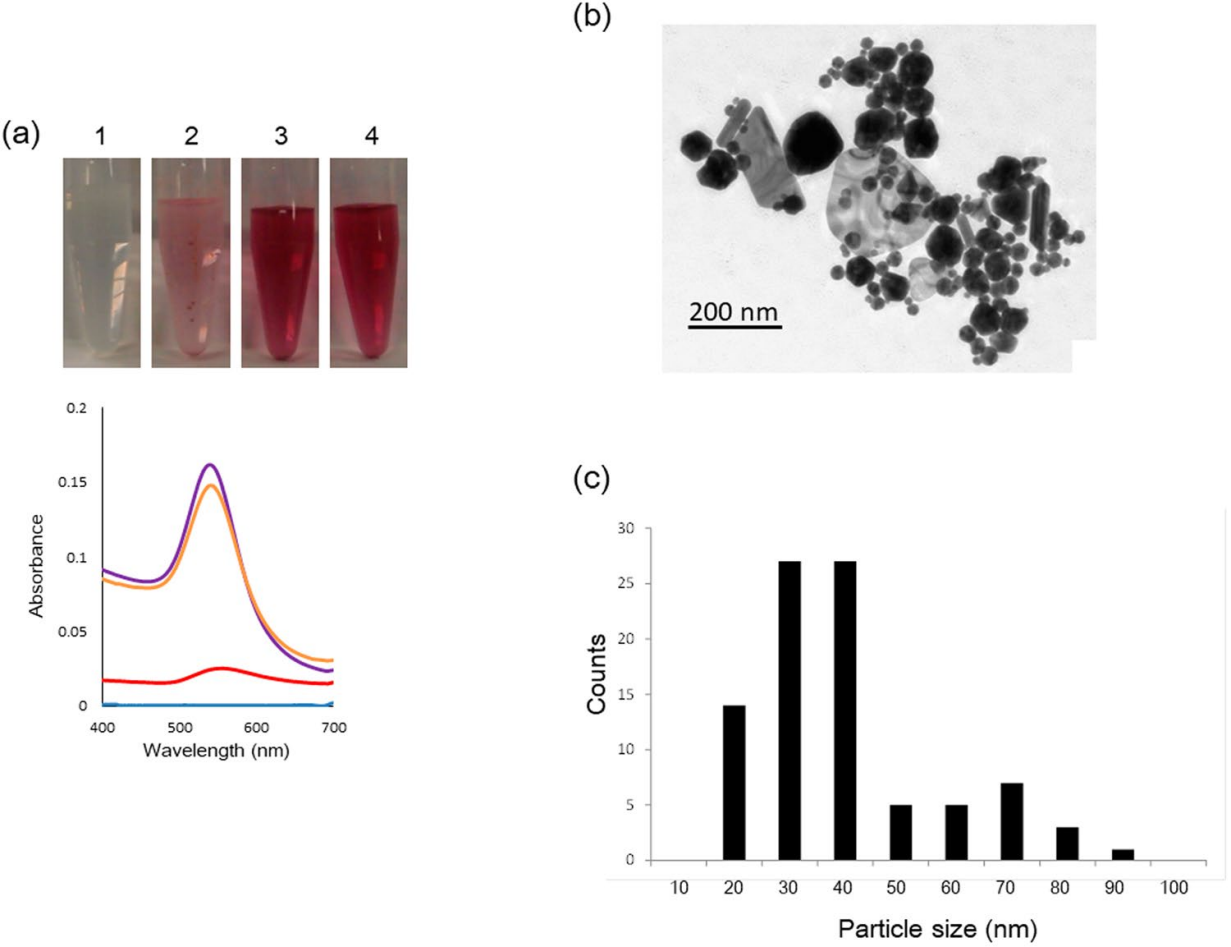
(**a**) Auric acid solution (0.5 mM K[AuCl4]) with caDGDG (1: 0 μg/mL, 2: 10 μg/mL, 3: 50 μg/mL, 4: 100 μg/mL) and their UV/VIS spectra after 24 h. The blue line: 0 mg/L DGDG, the red line: 10 μg/mL DGDG, the orange line: 50 μg/mL DGDG, the purple line: 100 μg/mL in UV/VIS spectra. (**b**) TEM image of gold nanoparticles synthesized by caDGDG (100 μg/mL). (**c**) The size distribution of nanoparticles in the image of (**b**).

