# Supplementary material for: Formation of gold nanoparticles by glycolipids of Lactobacillus casei
Source: Sci Rep. 2016 Oct 11;6:34626. doi: 10.1038/srep34626 (PMC5057074; doi:10.1038/srep34626)

Title

Formation of gold nanoparticles by glycolipids of *Lactobacillus casei*

Fumiya Kikuchi[1], Yugo Kato[1], Kazuo Furihata[1], Toshihiro Kogure[2], Yuki Imura[1], Etsuro Yoshimura[1,3*], Michio Suzuki[1*]

[1]Department of Applied Biological Chemistry, Graduate School of Agricultural and Life Sciences, The University of Tokyo, 1-1-1 Yayoi, Bunkyo-ku, Tokyo 113-8657, Japan.

[2]Department of Earth and Planetary Science, Graduate School of Science, The University of Tokyo, 7-3-1 Hongo, Bunkyo-ku, Tokyo 113-0033, Japan

[3]The Open University of Japan, 2-11 Wakaba, Mishima-ku, Chiba-city, Chiba, 261-8586, Japan

*To whom correspondence should be addressed. M. Suzuki, Tel: +81-3-5841-5156; E-mail: amichiwo@mail.ecc.u-tokyo.ac.jp, E. Yoshimura, Tel: +81-3-5841-5153; E-mail: ayoshim@mail.ecc.u-tokyo.ac.jp

Keywords

autoxidation, biomineralization, glycolipid, gold nanoparticle, *Lactobacillus casei*

Fig. S1. (a) Auric acid solution (1: 0 mM $K[AuCl_4]$, 2: 0.25 mM $K[AuCl_4]$, 3: 0.5 mM $K[AuCl_4]$) without *L. casei* cells and their UV/VIS spectra after 24 h. (b) Auric acid solution (1: 0 mM $K[AuCl_4]$, 2: 0.25 mM $K[AuCl_4]$, 3: 0.5 mM $K[AuCl_4]$) with *L. casei* cells (0.25 g/L) their UV/VIS spectra after 24 h. (c) Auric acid solution (1: 0 mM $K[AuCl_4]$, 2: 0.25 mM $K[AuCl_4]$, 3: 0.5 mM $K[AuCl_4]$) with *L. casei* cells (1.0 g/L) their UV/VIS spectra after 24 h. (d) Auric acid solution (1: 0 mM $K[AuCl_4]$, 2: 0.25 mM $K[AuCl_4]$, 3: 0.5 mM $K[AuCl_4]$) with *L. casei* cells (2.0 g/L) their UV/VIS spectra after 24 h. The green line: 0 mM $K[AuCl_4]$, the blue line: 0.25 mM $K[AuCl_4]$ and the red line: 0.5 mM $K[AuCl_4]$ in UV/VIS spectra.

Fig. S2. (a) Magnified image of the cell from *L .casei* treated with Au(+) solution. (b) The size distribution of nanoparticles in the image of (a).

Fig. S3. The visual of each solution in the cell. 1: Pure water, 2: the supernatant of Au(-) after ultrasonication. 3: the supernatant of Au(+) after ultrasonication.

Fig. S4. MALDI-TOF-MS spectra of Au(-) extract (a) and Au(+) extract (b).

Fig. S5. MALDI-TOF/TOF-MS spectra of 939.6 (a), 953.6 (b), 967.6 (c), 981.6 (d) and 969.3 (e) peaks.

Fig. S6. DQF-COSY spectrum of DGDG.

Fig. S7. HSQC spectrum of DGDG.

Fig. S8. HMBC spectrum of DGDG.

Fig. S9. The schematic representation of the correlations from DQF-COSY and HMBC.

Fig. S10. (a) MALDI-TOF-MS spectrum of the extract from spot 2 in Fig. 3 (c). (b) MALDI-TOF-MS analysis of Au(-) extract. (c) MALDI-TOF-MS analysis of Au(+) extract.

Fig. S11. MALDI-TOF/TOF-MS spectra of 1101.2 (a), 1115.2 (b), 1129.2 (c) and 1143.2 (d) peaks.

Fig. S12 (a) Auric acid solution (0.25 mM $K[AuCl_4]$) with DGDG purified from the TLC plate (1: 0 μg/mL, 2: 5.0 μg/mL, 3: 40 μg/mL, 4: 160 μg/mL) and their UV/VIS spectra after 24 h. The blue line: 0 mg/mL DGDG, the red line: 5.0 μg/mL DGDG, the green line: 40 μg/mL DGDG, the purple line: 160 μg/mL in UV/VIS spectra. (b) The size distribution of nanoparticles in the image of Fig. 4f.

Fig. S13 (a) Auric acid solution (0.5 mM $K[AuCl_4]$) with caDGDG (1: 0 μg/mL, 2: 10 μg/mL, 3: 50 μg/mL, 4: 100 μg/mL) and their UV/VIS spectra after 24 h. The blue line: 0 mg/L DGDG, the red line: 10 μg/mL DGDG, the orange line: 50 μg/mL DGDG, the purple line: 100 μg/mL in UV/VIS spectra. (b) TEM image of gold nanoparticles synthesized by caDGDG (100 μg/mL). (c) The size distribution of nanoparticles in the image of (b)

Fig. S14 The speculated schematic scheme of autoxidation reaction of glycolipids with unsaturated fatty acids in auric acid solution.

Fig. S15. The correlation between dry weight of cells and OD_{600}.

Fig. S1

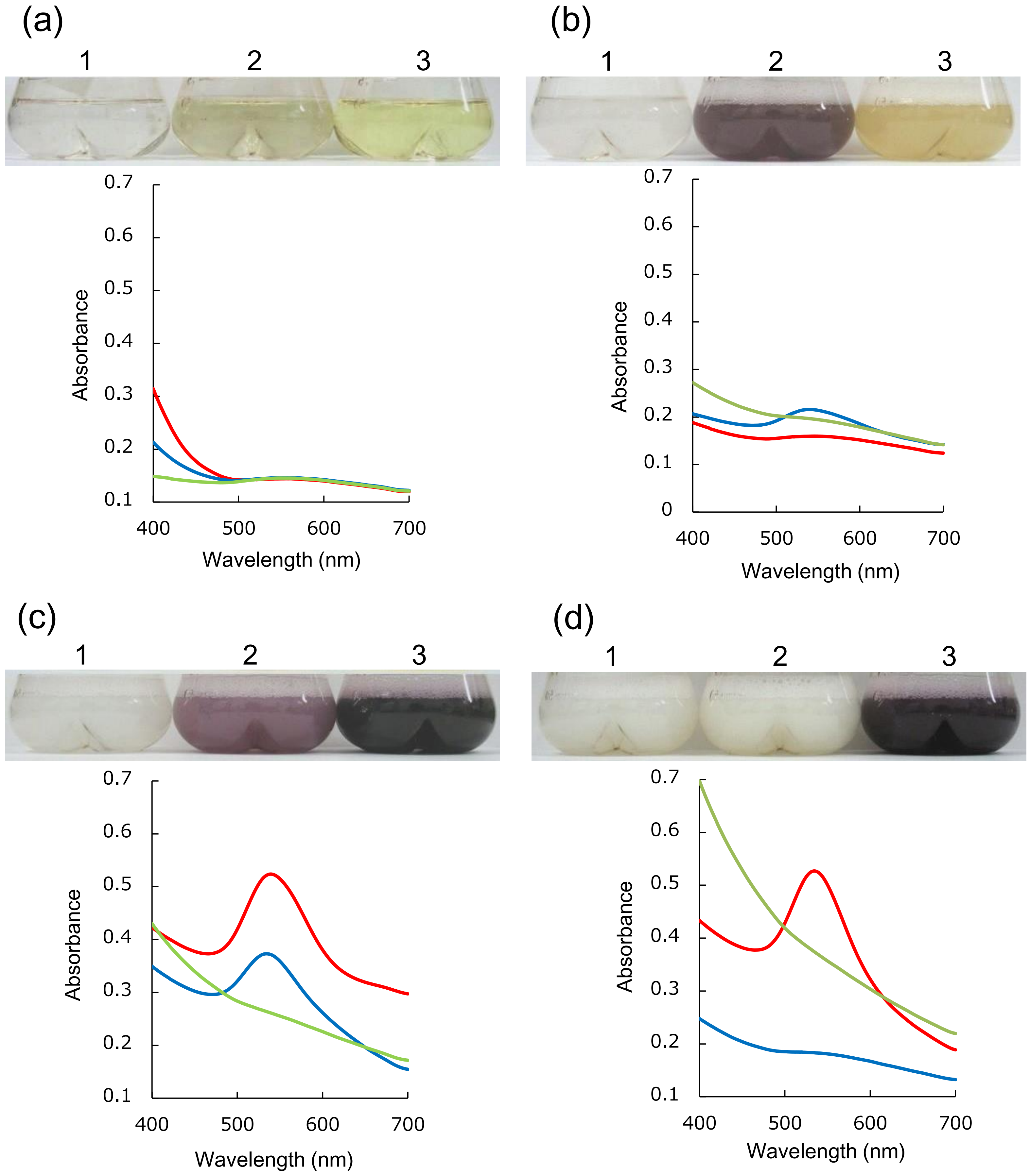
(a)
1
2
3
0.7
0.6
0.5
0.4
0.3
0.2
0.1
Absorbance
400
500
600
700
Wavelength (nm)
(b)
1
2
3
0.7
0.6
0.5
0.4
0.3
0.2
0.1
0
Absorbance
400
500
600
700
Wavelength (nm)
(c)
1
2
3
0.7
0.6
0.5
0.4
0.3
0.2
0.1
Absorbance
400
500
600
700
Wavelength (nm)
(d)
1
2
3
0.7
0.6
0.5
0.4
0.3
0.2
0.1
Absorbance
400
500
600
700
Wavelength (nm)

Fig. S2

(a)

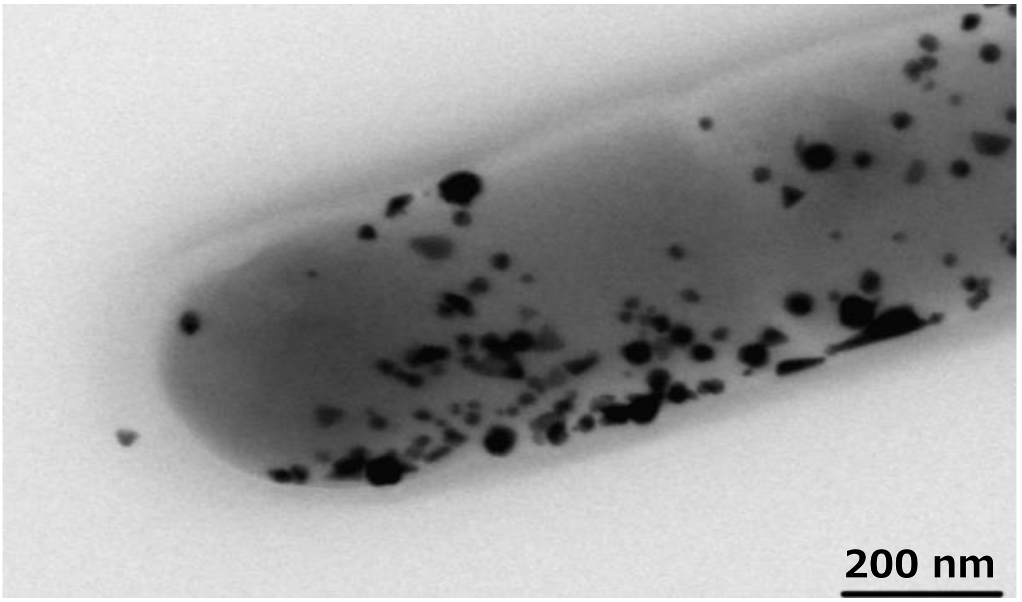

200 nm

(b)

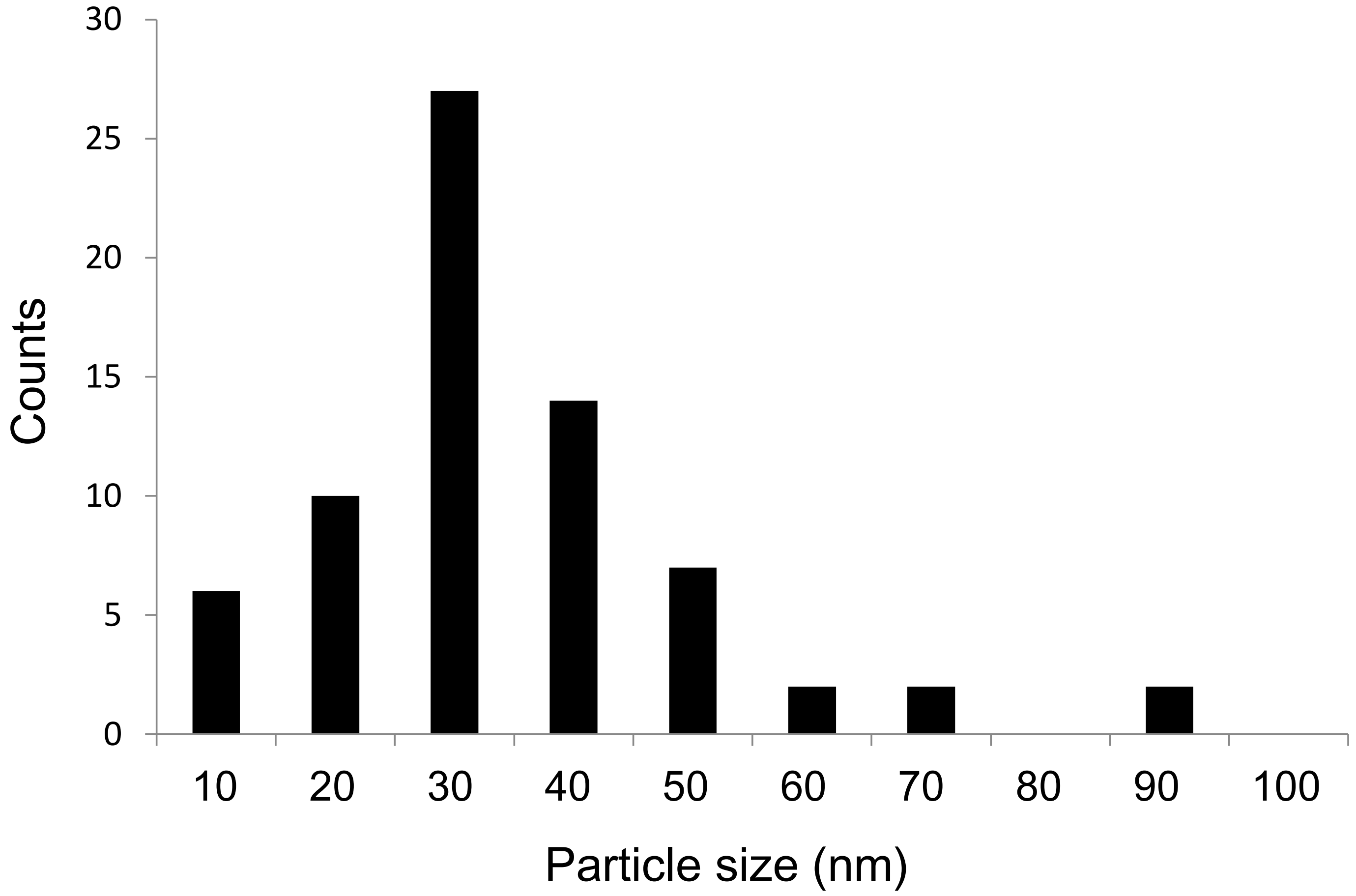

30
25
20
15
10
5
0
Counts
10
20
30
40
50
60
70
80
90
100
Particle size (nm)

Fig. S3

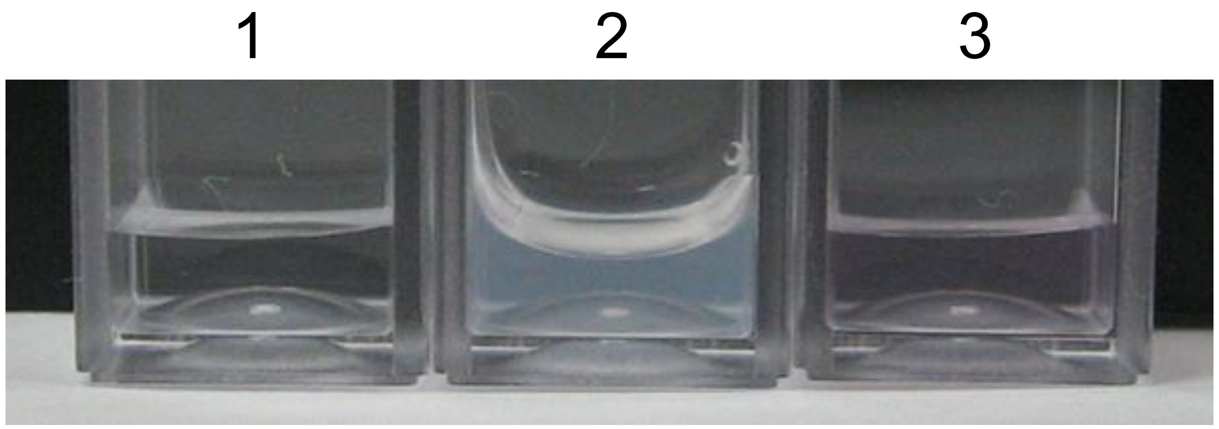

Fig. S4

(a)

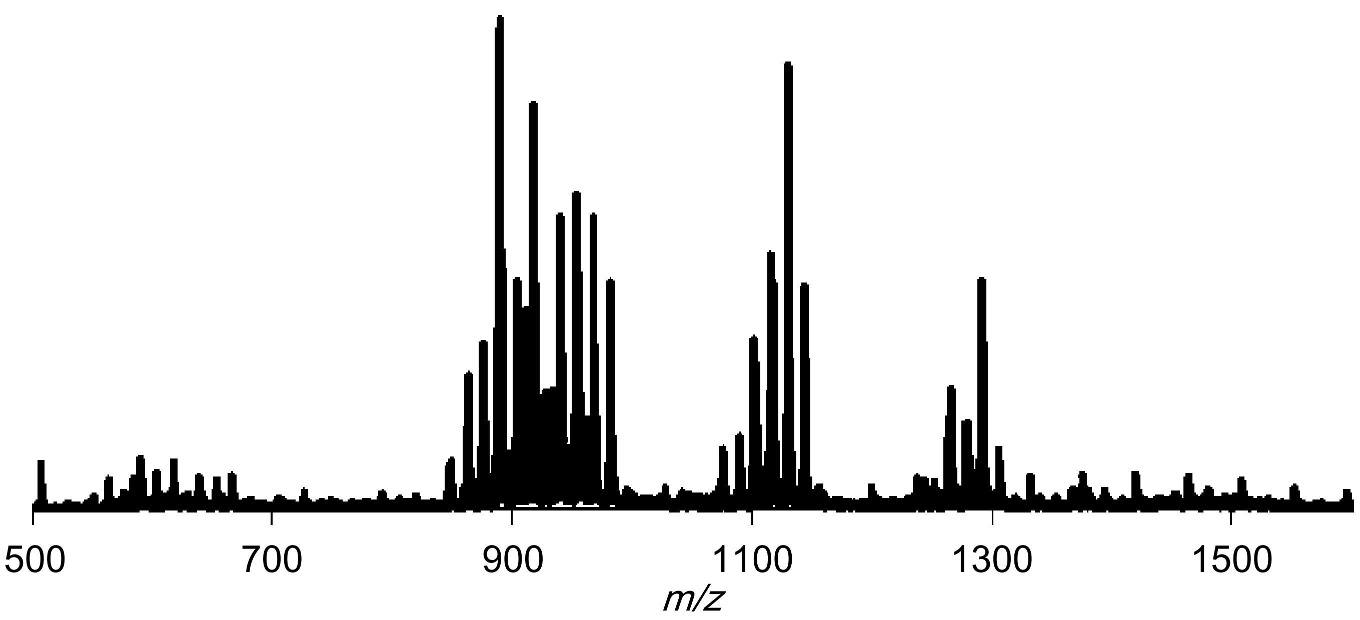

(b)

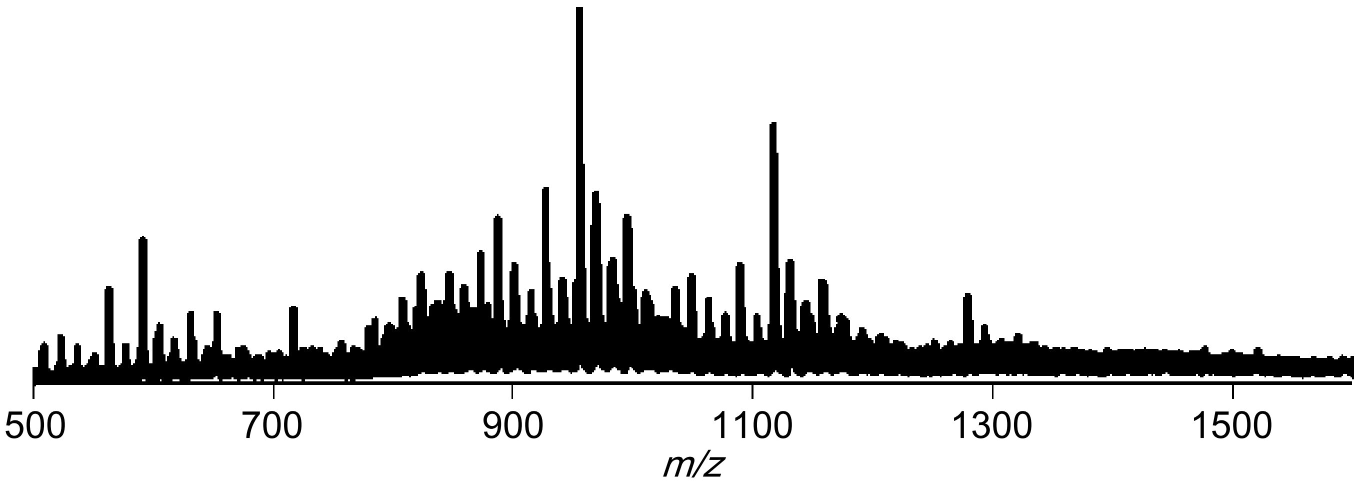

Fig. S5

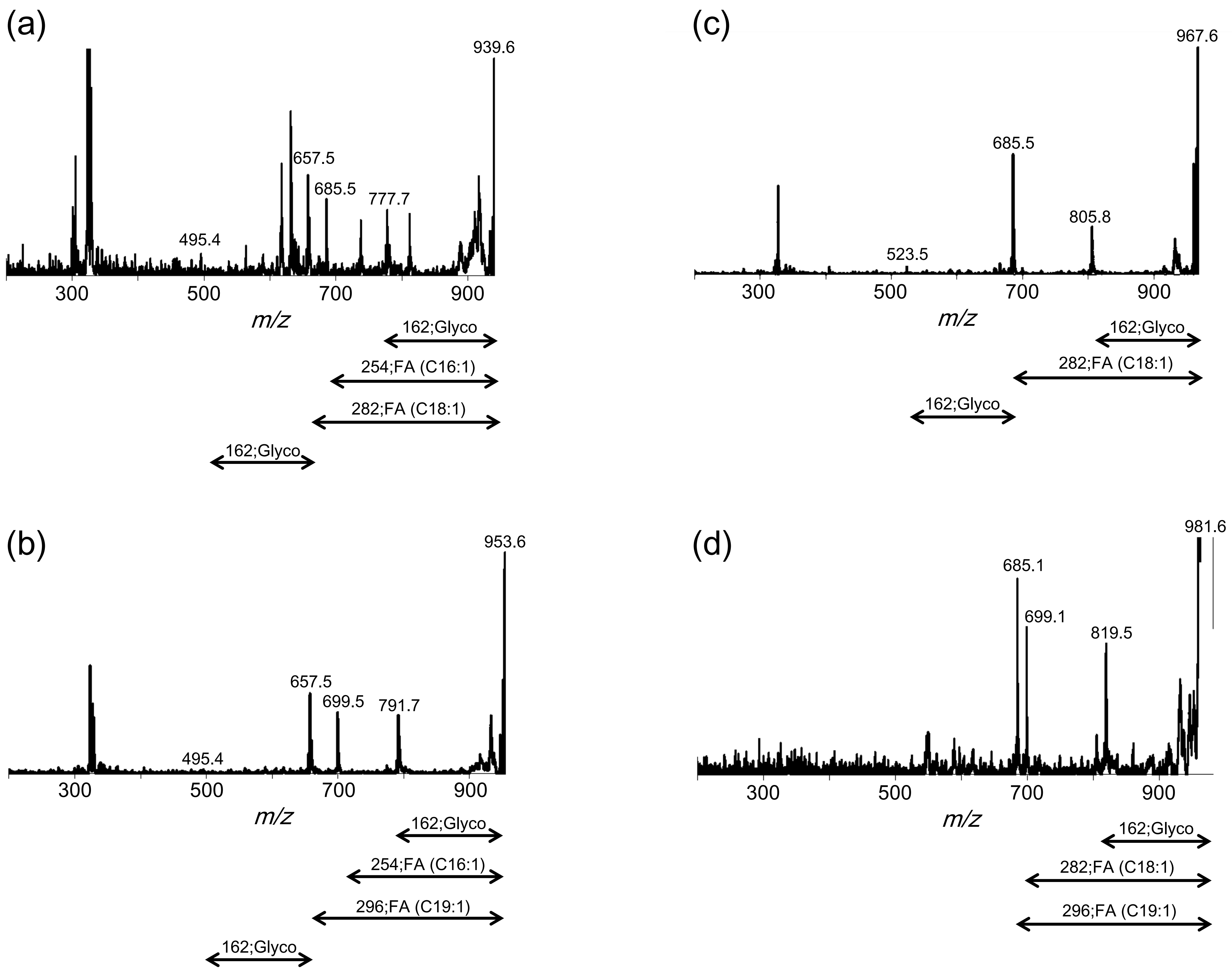

(a)
939.6
657.5
685.5
777.7
495.4
300
500
700
900
m/z
162;Glyco
254;FA (C16:1)
282;FA (C18:1)
162;Glyco
(b)
953.6
657.5
699.5
791.7
495.4
300
500
700
900
m/z
162;Glyco
254;FA (C16:1)
296;FA (C19:1)
162;Glyco
(c)
967.6
685.5
805.8
523.5
300
500
700
900
m/z
162;Glyco
282;FA (C18:1)
162;Glyco
(d)
981.6
685.1
699.1
819.5
300
500
700
900
m/z
162;Glyco
282;FA (C18:1)
296;FA (C19:1)

Fig. S5

(e)

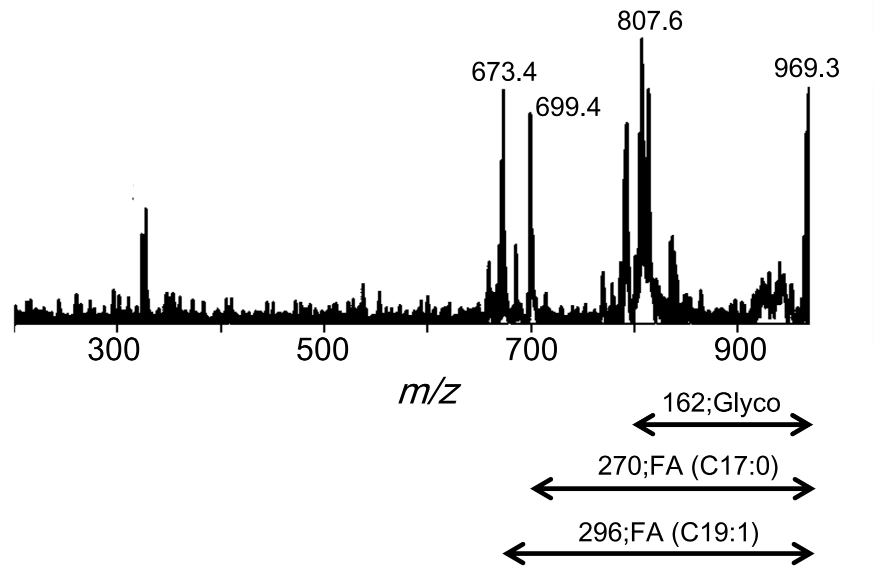

807.6
673.4
699.4
969.3
300
500
700
900
m/z
162;Glyco
270;FA (C17:0)
296;FA (C19:1)

Fig. S6

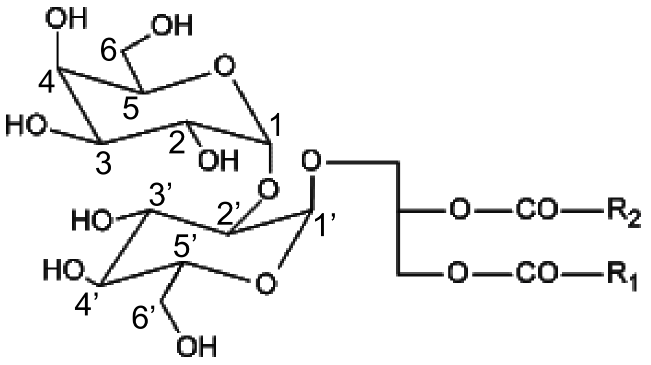
OH
OH
6
4
5
O
1
HO
2
3
OH
O
3'
2'
O
HO
1'
O—CO—R2
5'
HO
O
O—CO—R1
4'
6'
OH

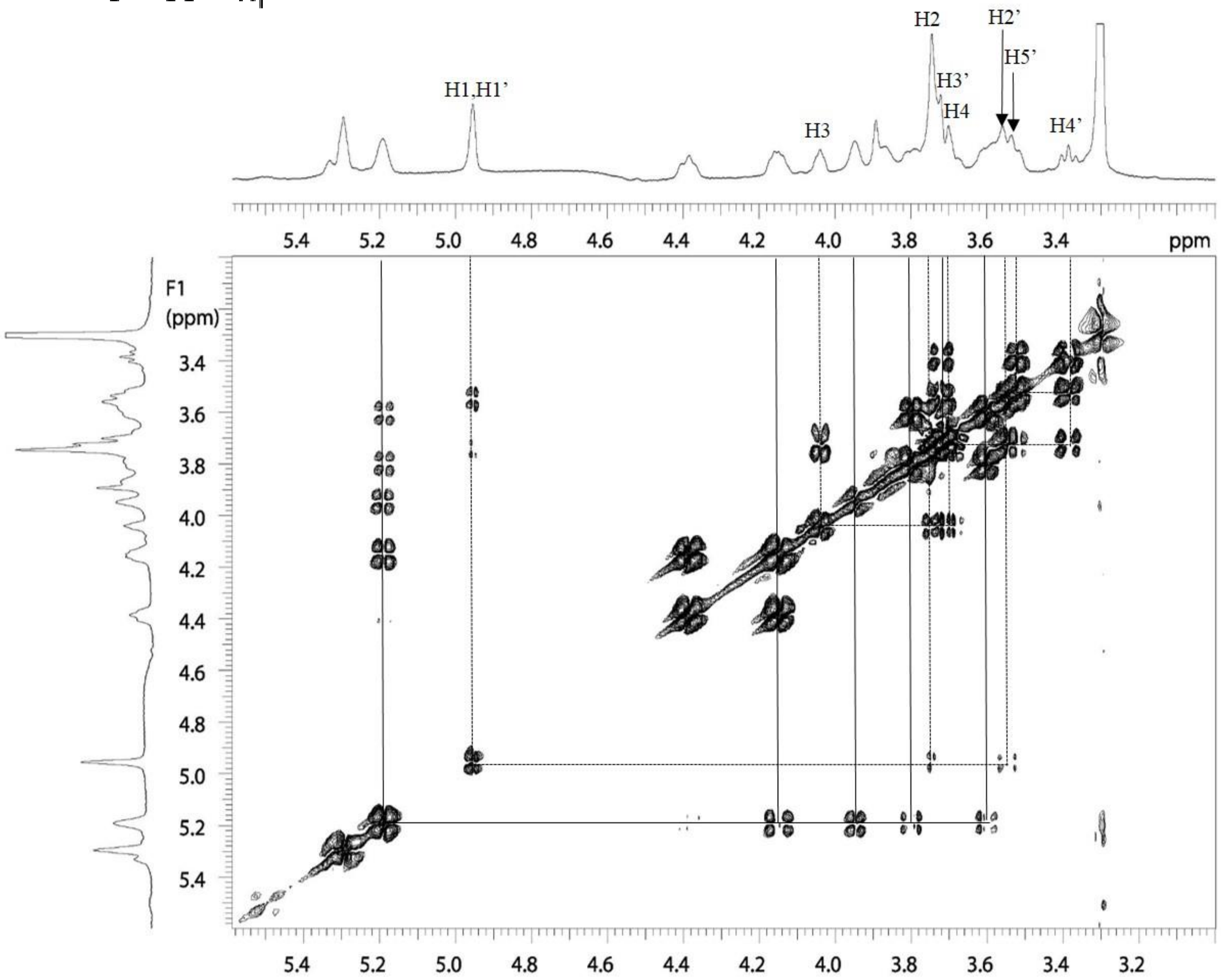
H1,H1'
H3
H2
H3'
H4
H2'
H5'
H4'
5.4
5.2
5.0
4.8
4.6
4.4
4.2
4.0
3.8
3.6
3.4
3.2
ppm
F1
(ppm)

Fig. S7

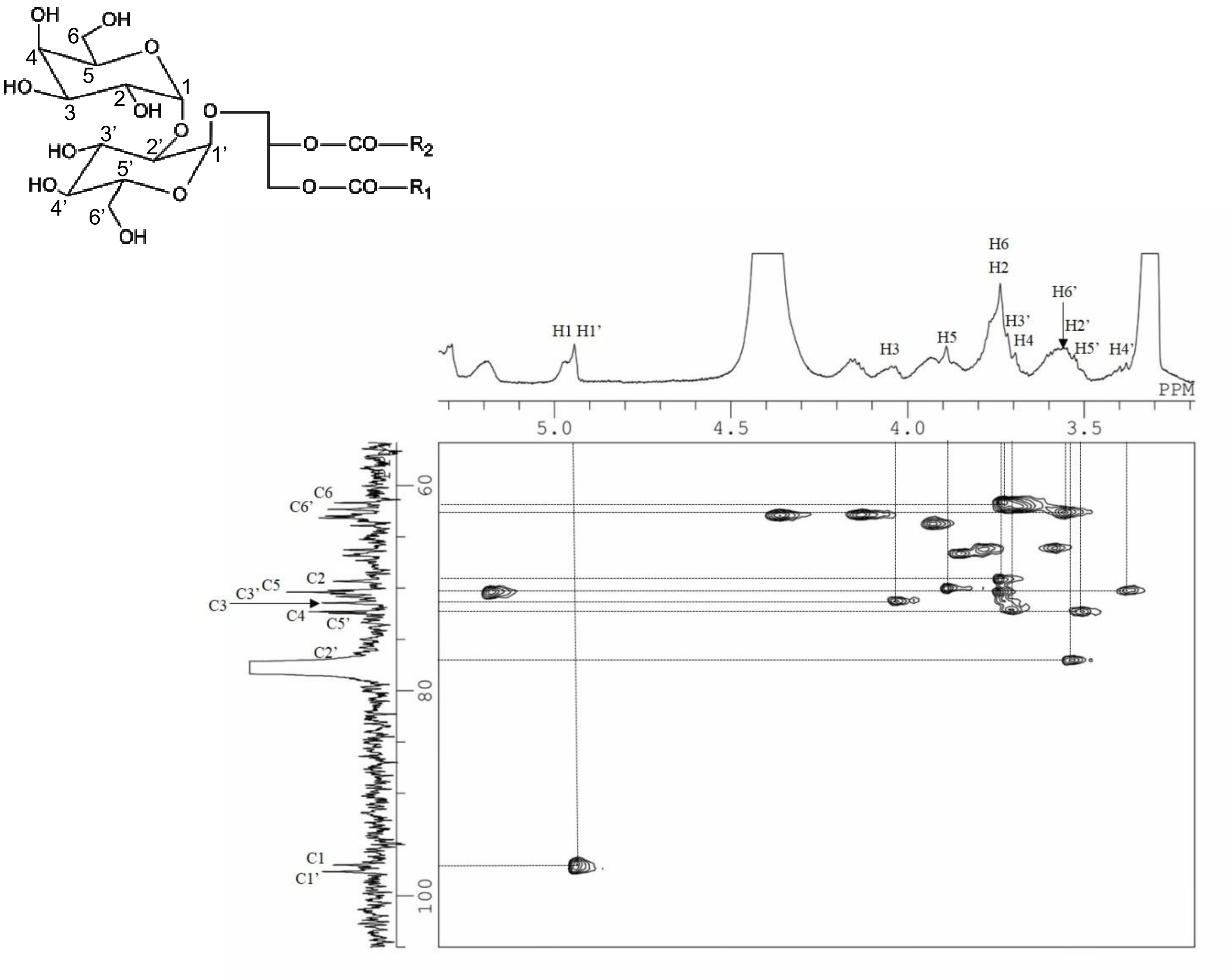
H1 H1'
H3
H5
H6
H2
H3'
H4
H6'
H2'
H5'
H4'
PPM
5.0
4.5
4.0
3.5
C6
C6'
C2
C5
C3'
C3
C4
C5'
C2'
C1
C1'
60
80
100

Fig. S8

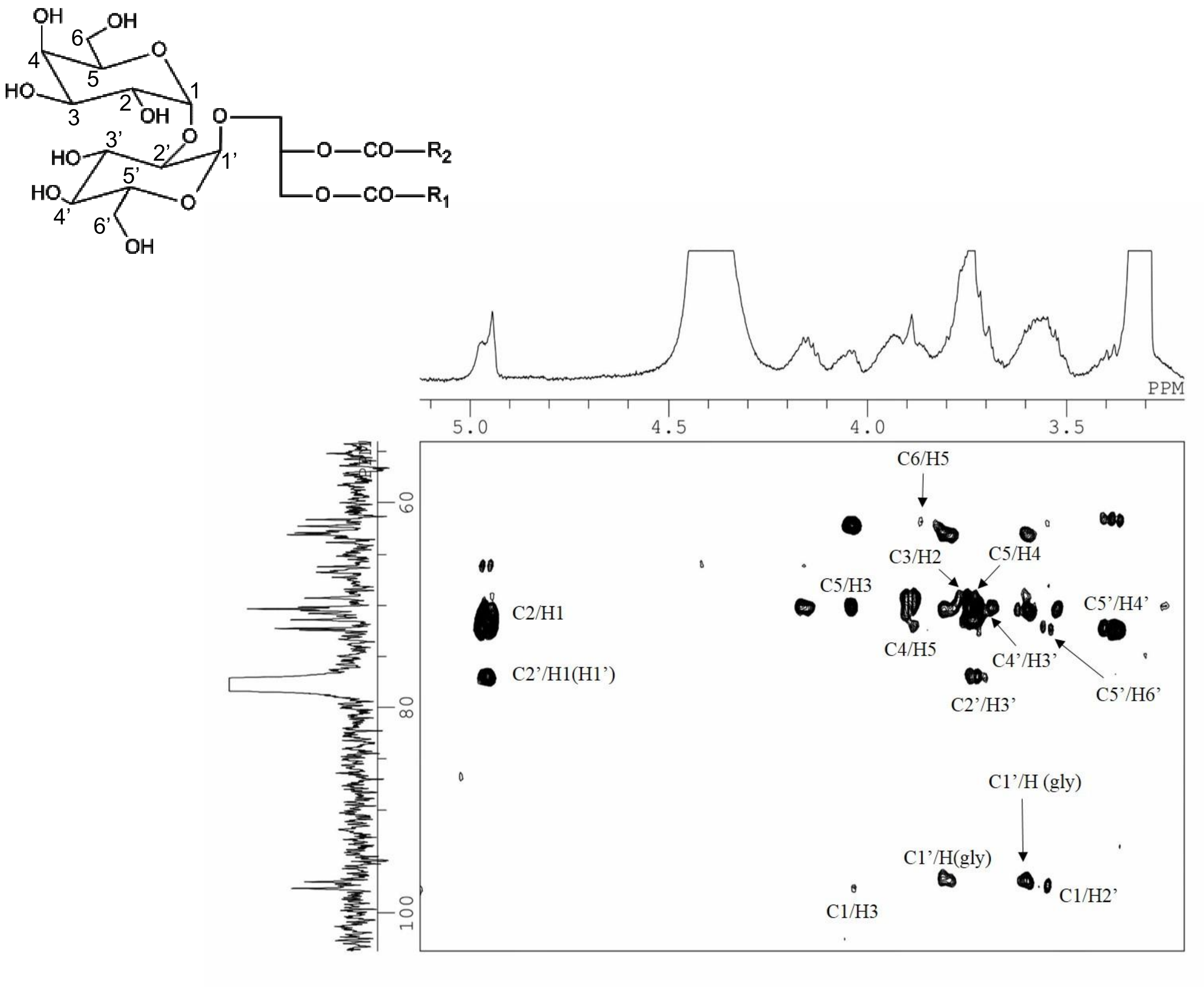

OH
OH
6
4
5
1
HO
2
OH
3
O
3'
2'
HO
1'
O—CO—R2
5'
HO
O—CO—R1
4'
6'
OH
PPM
5.0
4.5
4.0
3.5
60
80
100
C6/H5
C3/H2
C5/H4
C5/H3
C2/H1
C5'/H4'
C4/H5
C4'/H3'
C2'/H1(H1')
C5'/H6'
C2'/H3'
C1'/H (gly)
C1'/H(gly)
C1/H2'
C1/H3

Fig. S9

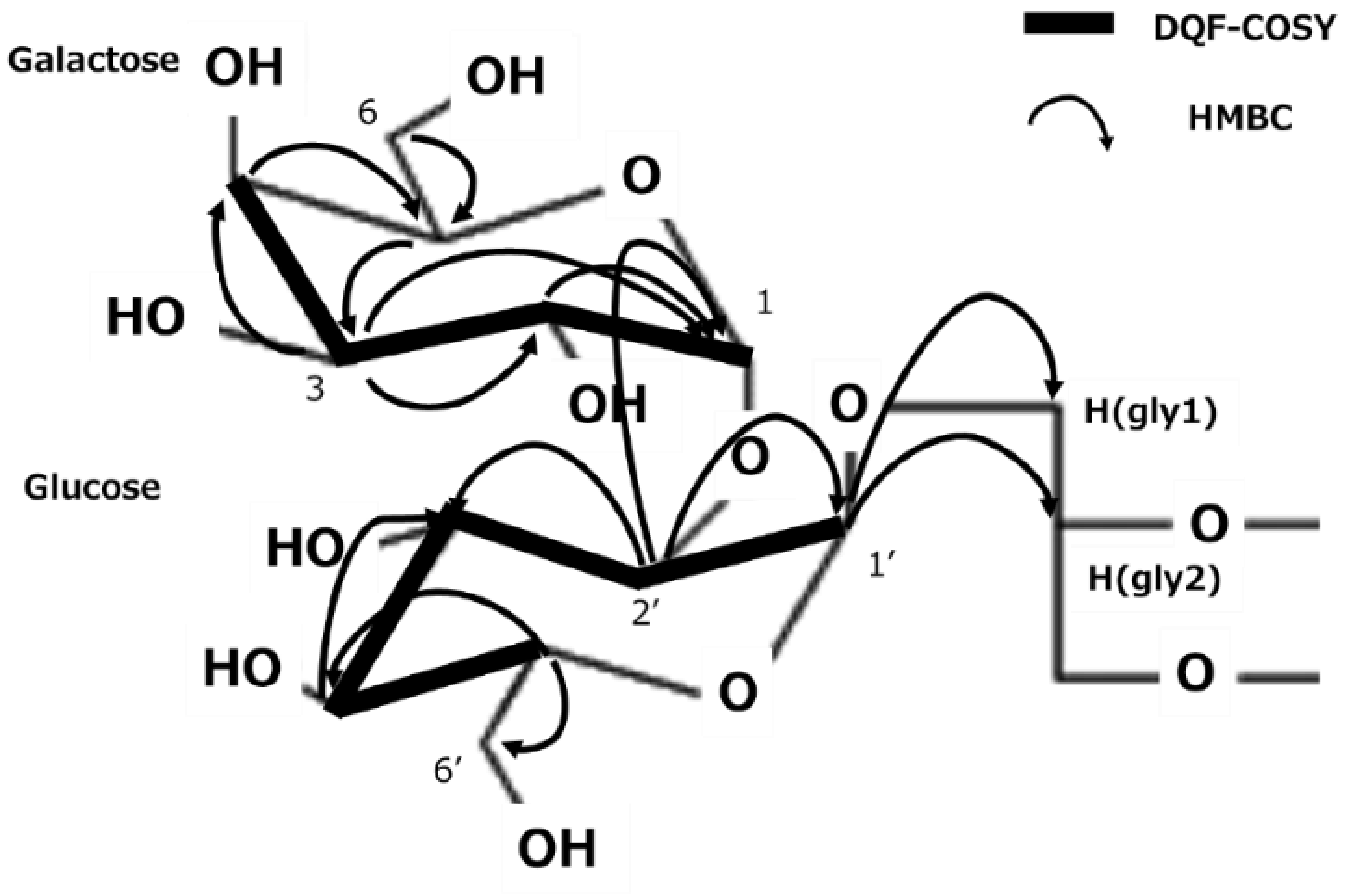

DQF-COSY
HMBC
Galactose
OH
6
OH
O
1
HO
3
OH
O
O
H(gly1)
Glucose
HO
2′
1′
O
H(gly2)
HO
O
O
6′
OH

Fig. S10

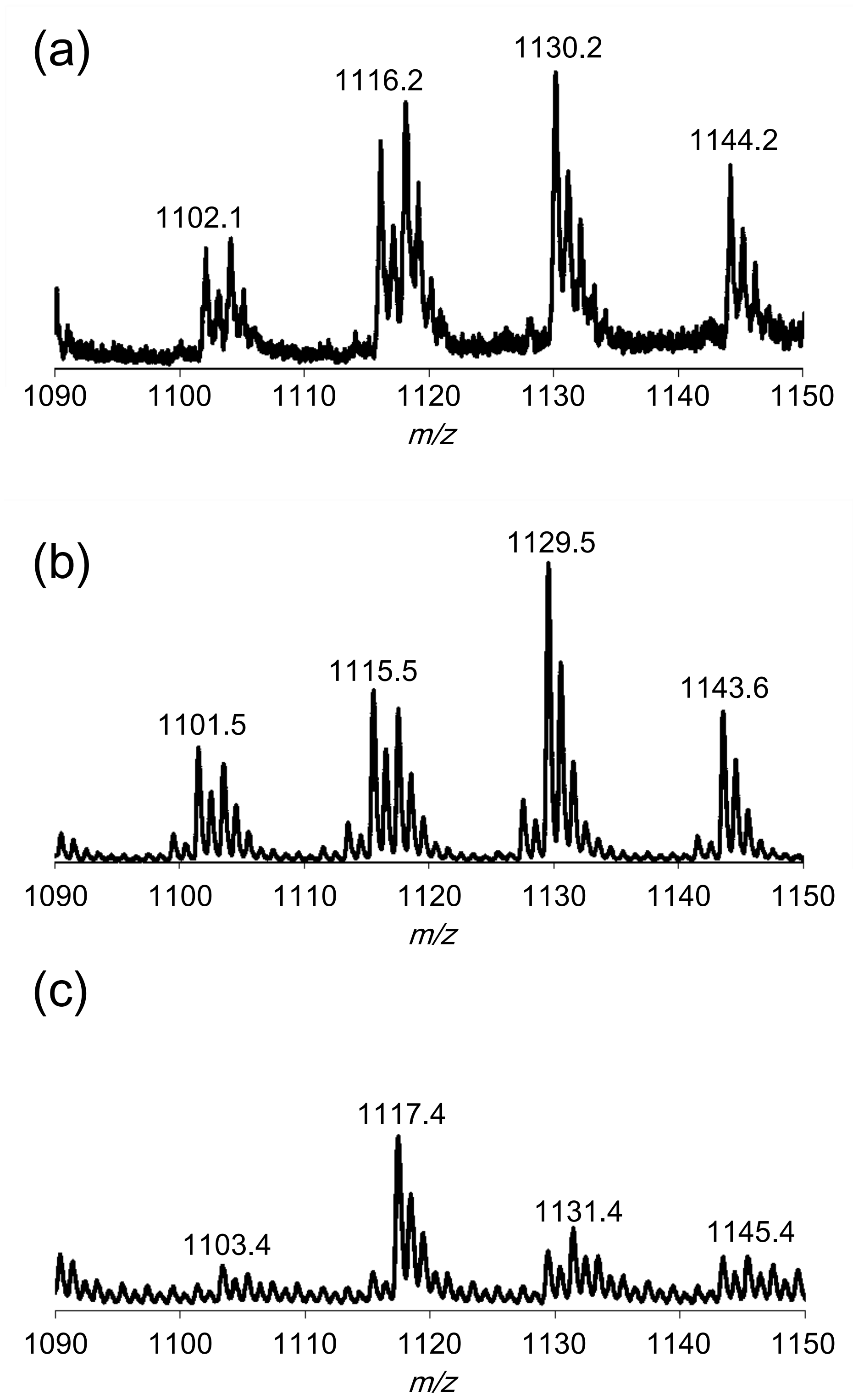
(a)
1102.1
1116.2
1130.2
1144.2
1090
1100
1110
1120
1130
1140
1150
m/z
(b)
1101.5
1115.5
1129.5
1143.6
1090
1100
1110
1120
1130
1140
1150
m/z
(c)
1103.4
1117.4
1131.4
1145.4
1090
1100
1110
1120
1130
1140
1150
m/z

Fig. S11

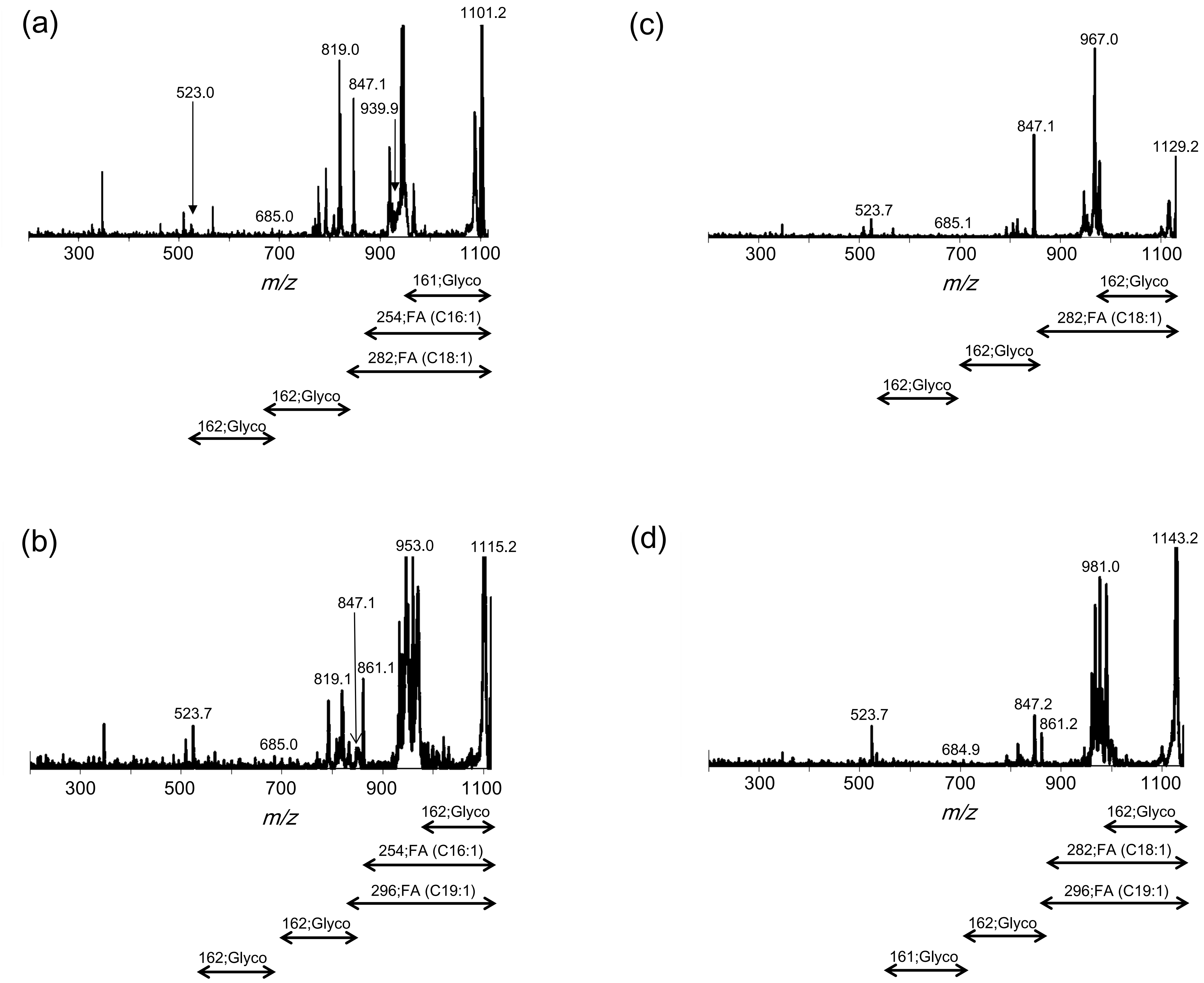

(a)
1101.2
819.0
847.1
939.9
523.0
685.0
300
500
700
900
1100
m/z
161;Glyco
254;FA (C16:1)
282;FA (C18:1)
162;Glyco
162;Glyco
(c)
967.0
847.1
1129.2
523.7
685.1
300
500
700
900
1100
m/z
162;Glyco
282;FA (C18:1)
162;Glyco
162;Glyco
(b)
953.0
1115.2
847.1
861.1
819.1
523.7
685.0
300
500
700
900
1100
m/z
162;Glyco
254;FA (C16:1)
296;FA (C19:1)
162;Glyco
162;Glyco
(d)
1143.2
981.0
847.2
861.2
523.7
684.9
300
500
700
900
1100
m/z
162;Glyco
282;FA (C18:1)
296;FA (C19:1)
162;Glyco
161;Glyco

Fig. S12

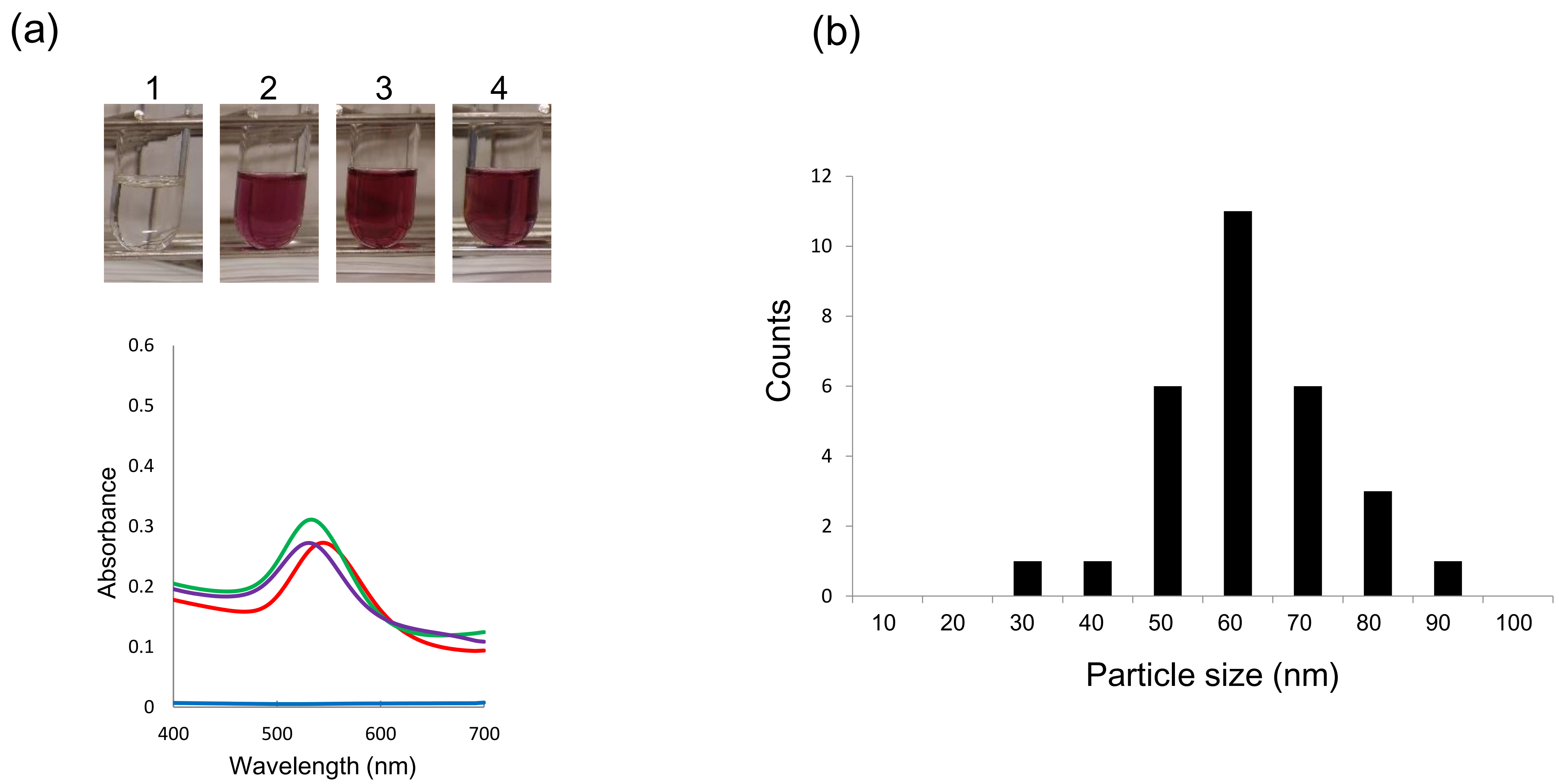

(a)
1
2
3
4
0.6
0.5
0.4
0.3
0.2
0.1
0
Absorbance
400
500
600
700
Wavelength (nm)
(b)
12
10
8
6
4
2
0
Counts
10
20
30
40
50
60
70
80
90
100
Particle size (nm)

Fig. S13

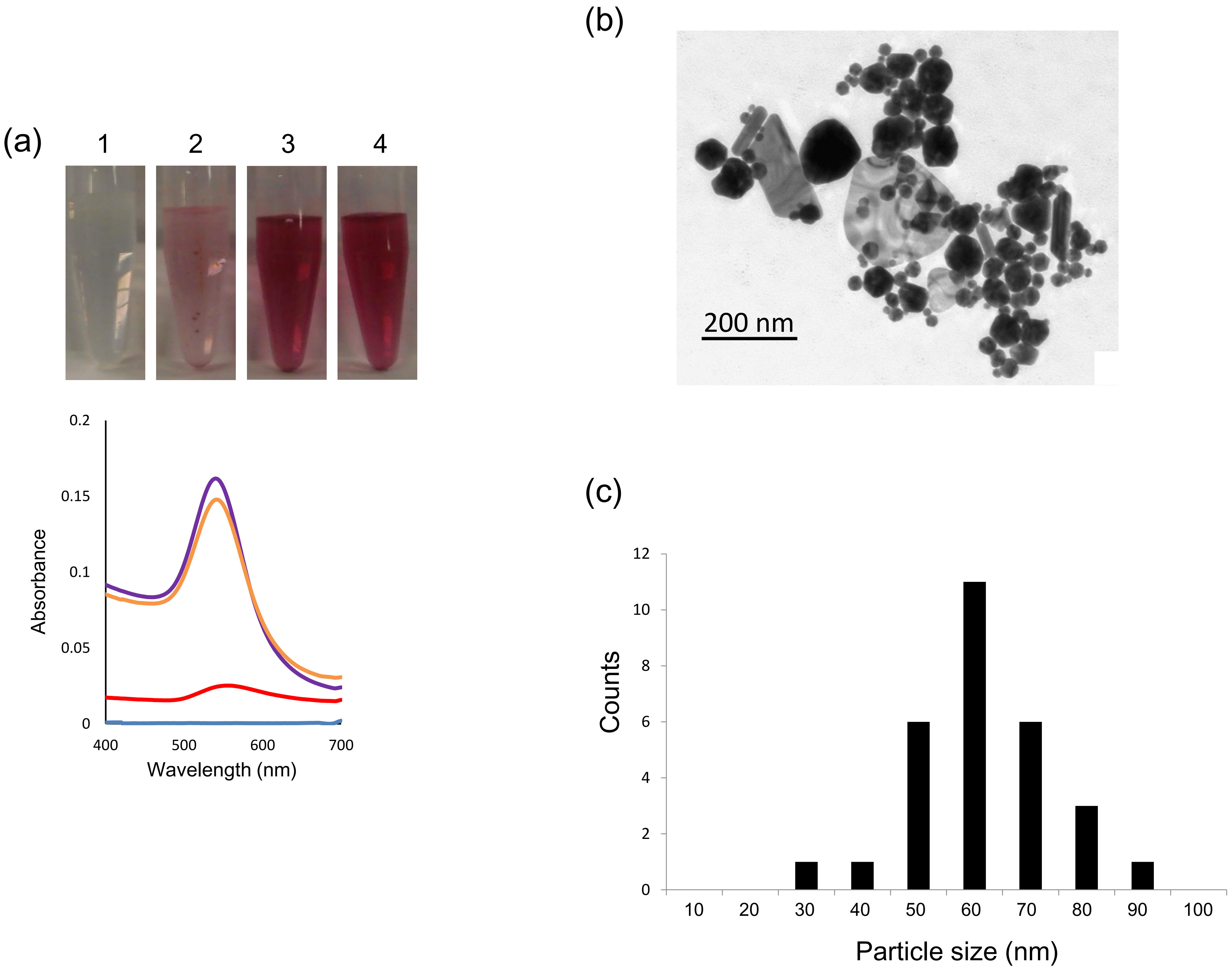
(a)
1
2
3
4
0.2
0.15
0.1
0.05
0
Absorbance
400
500
600
700
Wavelength (nm)
(b)
200 nm
(c)
12
10
8
6
4
2
0
Counts
10
20
30
40
50
60
70
80
90
100
Particle size (nm)

Fig. S14

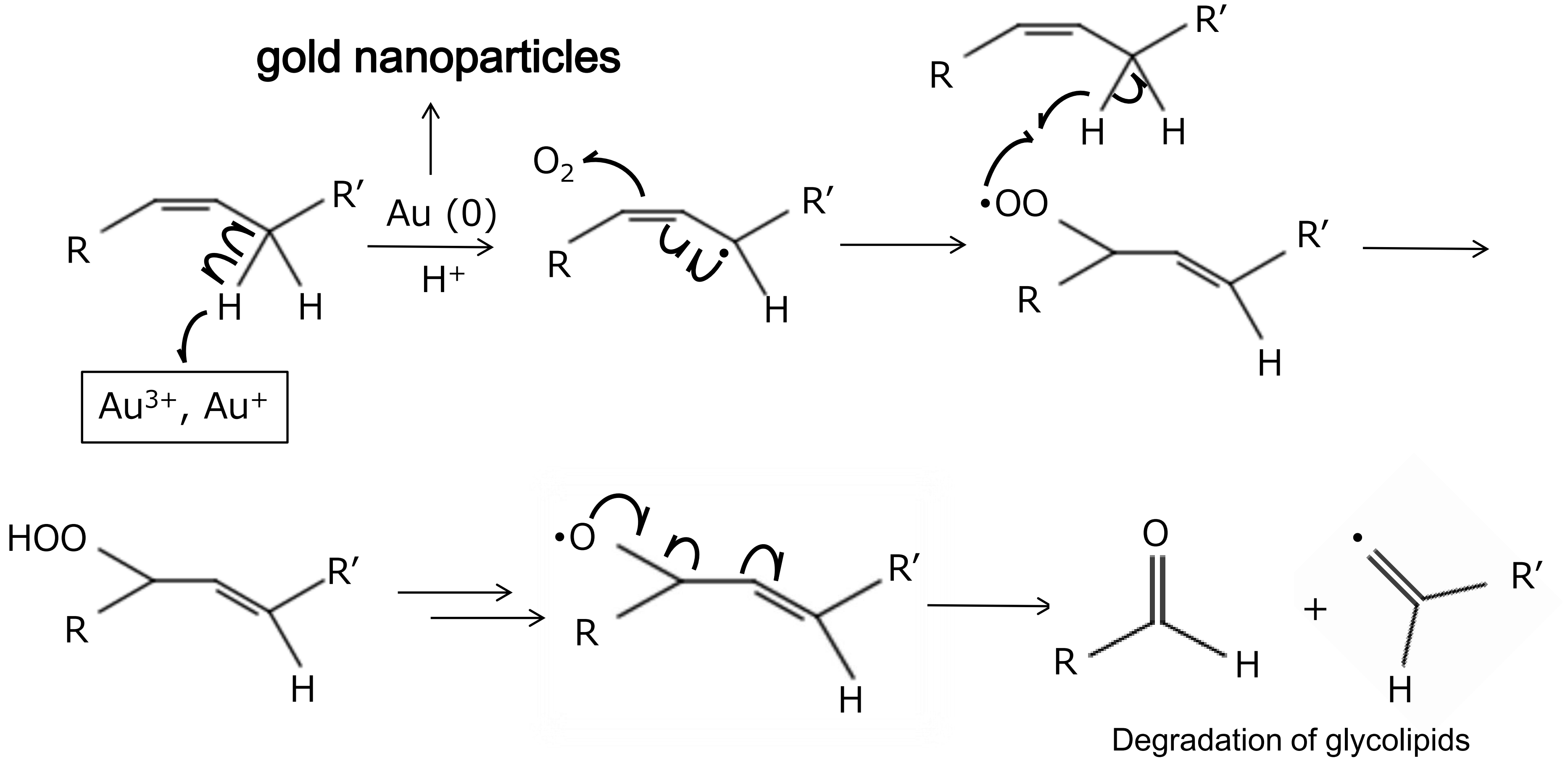

gold nanoparticles
Au (0)
H^+
Au^{3+}, Au^+
R
R′
H
H
O_2
•OO
HOO
•O
O
+
Degradation of glycolipids

Fig. S15

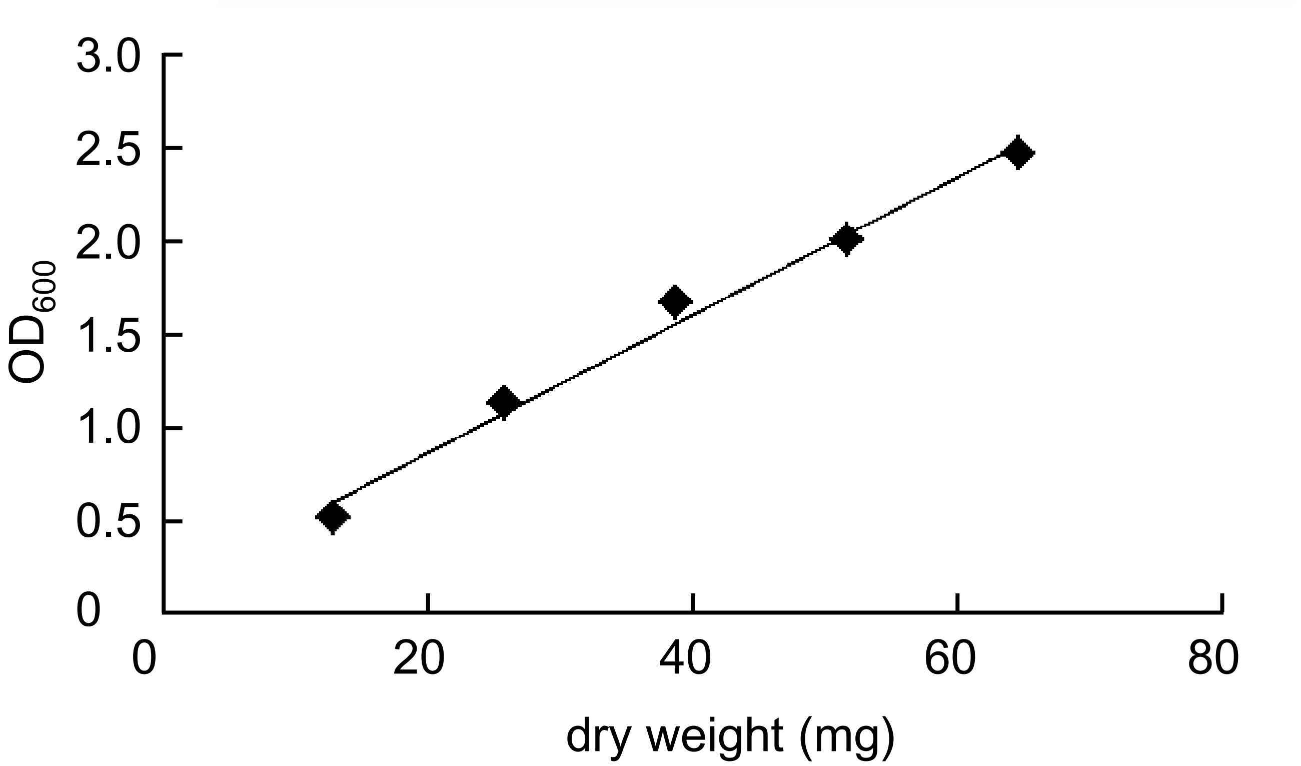

Supplement: Supplementary Information [file srep34626-s1.pdf]
